# *Lacticaseibacillus rhamnosus* Reduces the Pathogenicity of *Escherichia coli* in Chickens

**DOI:** 10.3389/fmicb.2021.664604

**Published:** 2021-06-01

**Authors:** Mengjiao Guo, Congyue Zhang, Chengcheng Zhang, Xiaorong Zhang, Yantao Wu

**Affiliations:** ^1^Jiangsu Co-Innovation Center for Prevention of Animal Infectious Diseases and Zoonoses, College of Veterinary Medicine, Yangzhou University, Yangzhou, China; ^2^Joint International Research Laboratory of Agriculture and Agri-Product Safety, Yangzhou University (JIRLAAPS), Yangzhou, China

**Keywords:** adhesion, intestinal microbiota, innate immune response, disease resistance, *Lacticaseibacillus rhamnosus*

## Abstract

*Lacticaseibacillus rhamnosus* is a recognized probiotic that is widely used in scientific research and clinical applications. This study found that the *Lacticaseibacillus rhamnosus* GG (LGG) strain can reduce the adhesion of *Escherichia coli* (*E. coli*) to primary chicken intestinal epithelial cells by 75.7% and inhibit 41.7% of the *E. coli* that adhere to intestinal epithelial cells. Additionally, LGG showed strong inhibitory ability on the growth of *E. coli*, *Staphylococcus aureus*, *Salmonella* Paratyphi B, and *Salmonella* Enteritidis *in vitro*. Furthermore, the influence of LGG on the growth performance, intestinal flora, immunity, and disease resistance of chickens was explored. Chickens fed with LGG exhibited increased average daily weight gain and concentrations of sIgA, IgG, and IgM than did controls. After 21 days of feeding, a diet with LGG increased the diversity of intestinal microbiota and maintained intestinal health. Moreover, LGG promoted immunologic barriers by upregulating cytokines and chemokines via the Toll-like receptor. The major pro-inflammatory factors, including *Myd88*, *NF-*κ*B*, *Il6*, and *Il8*, were upregulated compared to controls. After being challenged with *E. coli*, the survival rate of chickens fed with LGG was significantly higher than those in the control group, and decreased numbers of *E. coli* were detected in the heart and lungs of the LGG group. In summary, oral administration of LGG to chickens could improve growth performance, maintain intestinal homeostasis, and enhance innate immune response and disease resistance.

## Introduction

Administration of antibiotics is the most common treatment of bacterial diseases on livestock farms. Despite the improvement in growth performance, antibiotics have caused a number of problems (Simoneit et al., 2014). The administration of antibiotics can disrupt the balance of intestinal flora (Rashid et al., 2012), while excessive and unreasonable usage of antibiotics can generate drug-resistant bacteria (Lupindu et al., 2015). Therefore, a safe alternative to antibiotics is urgently needed. A dietary supplement of probiotics could improve growth performance, regulate immune response, prevent disease, and avoid a rise in drug resistance (Ng et al., 2009). In addition, administration of probiotics contributes to balancing intestinal microflora and reducing the oxygen in the intestines, which is essential for some pathogens (Pradhan et al., 2020). The intestinal microflora is closely correlated with the growth performance and health of the host; it plays an important role in regulating physiological function (Stanley et al., 2012) and can stimulate the development of intestinal cells, promote the absorption of nutrients, and regulate immunity (Abrams et al., 1963). Probiotics can improve immunity by influencing the intestinal microflora of chickens.

Probiotics like *Lactobacillus* and *Bacillus subtilis* have been widely used to promote immunity and prevent diseases in scientific research and clinical applications. The *Lacticaseibacillus rhamnosus* GG (LGG) strain can produce short-chain fatty acids, promoting intestinal health and improving immunity. LGG has been shown to improve growth performance, treat and prevent diarrhea, and act as an antioxidant (Wang et al., 2019). It is successfully used to prevent and treat human diarrhea, dental caries, and other diseases. Recently, it was confirmed that the metabolites of LGG promote the formation of intestinal mechanical barriers and immune barriers in newborn rats, as well as enhancing resistance to *Escherichia coli* (*E. coli*) infections (He et al., 2017). Moreover, LGG can produce 92 kinds of proteins under acidic conditions, most of which are related to forming biofilms, maintaining the structure of cell membranes, and regulating immune responses (Savijoki et al., 2011). Among these, the SpaC protein is an essential protein for LGG to adhere to the intestinal mucosa and to induce epithelial cells that produce reactive oxygen species (Yan et al., 2013). The novel protein HM0539 shows a potent protective effect on the intestinal barrier (Gao et al., 2019).

Innate immunity is the first line of defense against the colonization of pathogens, and it plays a critical role in the pathogenesis and progression of intestinal disorders. Previous research has shown that administration of probiotics could upregulate the expressions of Toll-like receptors (TLRs) and activate an innate immune response (Grabig et al., 2006). To gain better insight into the role of LGG on chickens, the probiotic effects of LGG were assessed. The adhesion ability and inhibitory effects on pathogenic bacteria were evaluated *in vitro*. The growth performance, the intestinal homeostasis, the immune response, and the disease resistance were explored in chickens.

## Materials and Methods

### Bacteria

*Lacticaseibacillus rhamnosus* GG (ATCC 53103) was obtained from the American Type Culture Collection and was cultured in MRS broth at 37°C. The bacterial pathogens of *Staphylococcus aureus* (ATCC 25923), *Salmonella* Paratyphi B (CMCC 50094), and *Salmonella* Enteritidis (CICC 24119) were stored in our laboratory and cultured in LB broth at 37°C. O1 *E. coli* was isolated from clinically infected ducks suffering from colibacillosis and stored in our laboratory.

### Primary Culture of Chicken Intestinal Epithelial Cells

Primary duodenal intestinal epithelial cells were prepared from 19-day-old specific pathogen-free chicken embryos as described previously (Guo et al., 2015; Zhang et al., 2019). The embryos were dissected, and the duodenal intestines were transferred to Hank’s Balanced Salt Solution (HBSS) supplemented with 100 U/mL of penicillin and streptomycin. Then the duodenal intestines were cut into small pieces and washed three times with HBSS. Thereafter, the duodenal intestines were digested with collagenase I (1 mg/mL) for 50 min at 37°C under steady agitation. The cell pellets were centrifuged at 800 rpm for 10 min and washed twice with HBSS. The cell pellets were resuspended in DMEM/F12 medium supplemented with epidermal growth factor (20 ng/mL), heparin sodium salt (100 μg/mL), insulin (5 μg/mL), and 2.5% fetal bovine serum. The larger pieces were filtered with a 200-mesh sieve. Fibroblasts and macrophages were discarded by 2 h adherence. Then, non-adherent cells were transferred to a new dish and incubated for 48 h.

### Screening for Probiotic Properties *in vitro*

#### Antimicrobial Activities

The antibacterial experiment followed the method described by Zhang D. et al. (2012) and Hwanhlem et al. (2017). The pathogenic bacteria (*S. aureus*, *S.* Paratyphi B, *S.* Enteritidis, and O1 *E. coli*) were adjusted to 10^6^ CFU mL^–1^ after overnight cultivation. The pathogenic bacteria were spread onto the LB agar plates. The LGG culture (OD_600_ = 1.0) was centrifuged at 3,000 rpm for 10 min to obtain the culture supernatant of LGG. After centrifugation, the LGG cells were washed twice with PBS and resuspended. In addition, the LGG culture (OD_600_ = 1.0) was frozen and thawed twice, then ultrasonically broken, and centrifuged at 3,000 rpm for 10 min to obtain the lysate. The LGG culture, lysate, culture supernatant, and bacterial cells were prepared and transferred to holes (approximately 5 mm in diameter) punched into the LB plates. Then, the LB agar plates were incubated at 37°C for 24 h. The antibacterial activities were determined by the size of the inhibition zone. Three replicates per sample were performed.

#### LGG Adhesion

Investigation of the adhesion of the LGG to primary chicken intestinal epithelial cells followed the method described by Fernández de Palencia et al. (2008). The LGG and *E. coli* cells were added to chicken intestinal epithelial cells (MOI = 100:1); then, the cells were incubated at 37°C and 5% CO_2_ for 1 h. Unbound bacteria were softly washed away with PBS; then, the cells were lyzed with 1% Triton X-100 for 20 min and spread onto MRS agar plates for a viable count. Three replicates per sample were performed.

To test the competition between LGG and *E. coli* for cell adhesion, LGG and *E. coli* (1:1) were simultaneously added to chicken intestinal epithelial cells and incubated for 1 h. Unbound bacteria were removed, and *E. coli* counts were carried out. The ability of LGG to inhibit the adhesion of *E. coli* was assessed as follows: LGG was first added to intestinal epithelial cells and incubated for 1 h, after which unbound LGG was removed, and *E. coli* was added to the wells. After incubation for 1 h, adherent *E. coli* was counted. To test the ability of LGG to displace previously adhered *E. coli*, *E. coli* was first added to intestinal epithelial cells and incubated for 1 h, after which unbound *E. coli* was removed and LGG was added to the wells. After incubation for 1 h, adherent *E. coli* was counted.

### Animal Experiments

Healthy newborn Ross 308 chickens were raised under the same conditions with sufficient water and food, and randomly allotted to two groups (basal diet or supplemented with 10^6^ CFU g^–1^ LGG). The composition and nutrient levels of the basal diets are shown in Table 1. Each group consisted of three replicates with 30 chickens per replicate. The chickens were weighed individually, and blood samples were collected from the wing vein at days 14 and 21. On day 21, five chickens per group were randomly selected and euthanized. The fecal contents, blood, spleens, and livers were collected. IgG, IgM, and sIgA in serum were detected by using chicken Immunoglobulin G ELISA kit, chicken Immunoglobulin M ELISA kit, and chicken secretory Immunoglobulin A ELISA kit (SenBeiJia, Nanjing, China), respectively. The remaining chickens were challenged with 10^6^ CFU *E. coli.* Five chickens per group were randomly selected and euthanized at 1 and 3 days post-infection (dpi). The hearts, livers, spleens, lungs, and kidneys were collected.

**TABLE 1:** Composition and nutrient levels of basal diets (air-dry basis).

Ingredients	Content	Nutrient levels	Content
Corn	50.75	ME/(MJ/kg)	11.3
Soybean meal	32.94	CP	19.06
Fish meal	2.01	Met	0.50
CaHPO_4_	1.92	Lys	1.15
Limestone	1.25	Ca	1.00
Corn protein flour	4.83		
NaCl	0.30		
Wheat	5.00		
Premix	1		
Total	100		

*Per kg of premix: Vitamin A, 10,000 IU; Vitamin B_1_, 6.0 mg; Vitamin B_12_, 40.0 mg; Vitamin D_3_, 7,000 IU; Vitamin E, 50 IU; Vitamin B_7_, 2.0 mg; Vitamin B_3_, 32.5 mg; Cu, 8.0 mg; Fe, 90.0 mg; Mn, 70.0 mg; Zn, 90.0 mg; I, 1.1 mg; Se, 0.2 mg.*

All experiments were carried out in accordance with the principles of the Basal Declaration and Recommendations of Committee on the Ethics of Animal Experiments of Yangzhou University, and the protocol was approved by the Committee on the Ethics of Animal Experiments of Yangzhou University.

### 16S rRNA Sequencing and Analysis

The DNA in the fecal content was extracted using HiPure Stool DNA Kit B (Magen, Shanghai, China), according to the manufacturer’s protocols. The highly variable regions of V3 and V4 on 16S rRNA were amplified using 20–30 ng DNA as templates (F: CCTACGGRRBGCASCAGKVRVGAAT; R: GGACTACNVGGGTWTCTAATCC). Illumina MiSeq/NovaSeq was used for two-terminal sequencing; the sequenced reads were spliced and filtered, and the chimera was removed. Obtained sequences were performed with operational taxonomic units (OTUs) clustering and diversity analysis.

### Quantitative Real-Time PCR

Total RNA was extracted from the spleens and livers using TRIzon Reagent (CoWin Biosciences, Beijing, China), and 1 μg of the total RNA was reverse-transcribed according to the instructions of the TranScript all-in-one first-strand cDNA Synthesis Supermix kit (Transgen, Beijing, China). The TransStartR Tip Green qPCR SuperMix kit (Transgen, Beijing, China) was used for qRT-PCR. The primer sequences used for qRT-PCR are listed in Table 2. The qRT-PCR was conducted on a total volume of 20 μL, and the amplification steps consisted of 94°C for 30 s, 40 cycles of denaturation at 94°C for 5 s, and extension 60°C for 34 s, as well as a dissociation curve analysis. The 2^–ΔΔCT^ method was used to estimate mRNA abundance. Relative gene expression levels were normalized by the housekeeping gene β-actin.

**TABLE 2:** Primers used in this study.

Primer name	Sequence (5′–3′)
*Mhc II*α-F	TGGGATCCTCCGTCCTGAAGCCGCAC
*Mhc II* α-R	GCGTCGACTCAGAGCAGCCCCGGTT
*Myd88*-F	TGATGCCTTCATCTGCTACTG
*Myd88*-R	TCCCTCCGACACCTTCTTTCTA
*NF-*κ*b*-F	CAGCCCATCTATGACAACCG
*NF-*κ*b*-R	TCCCTGCGTCTCCTCTGTGA
*Il1*β-F	GTGAGGCTCAACATTGCGCTGTA
*Il1*β-R	TGTCCAGGCGGTAGAAGATGAAG
*Il8*-F	ATGAACGGCAAGCTTGGAGCTG
*Il8*-R	TCCAAGCACACCTCTCTTCCATCC
*Ifn-*α-F	ATGCCACCTTCTCTCACGAC
*Ifn-*α-R	AGGCGCTGTAATCGTTGTCT
*Il6*-F	TCTGTTCGCCTTTCAGACCTA
*Il6*-R	GACCACCTCATCGGGATTTAT
*Tlr4*-F	AGTCTGAAATTGCTGAGCTCAAAT
*Tlr4*-R	GCGACGTTAAGCCATGGAAG
β*-actin*-F	GAGAAATTGTGCGTGACATCA
β*-actin*-R	CCTGAACCTCTCATTGCCA

### Statistical Analysis

The Student’s *t*-test was used to identify significant differences between experimental groups using the SPSS computer software (SPSS, Chicago, IL, United States). A *p*-value of < 0.05 was considered the threshold for statistical significance.

## Results

### Antibacterial Activity of LGG

**FIGURE 1:**
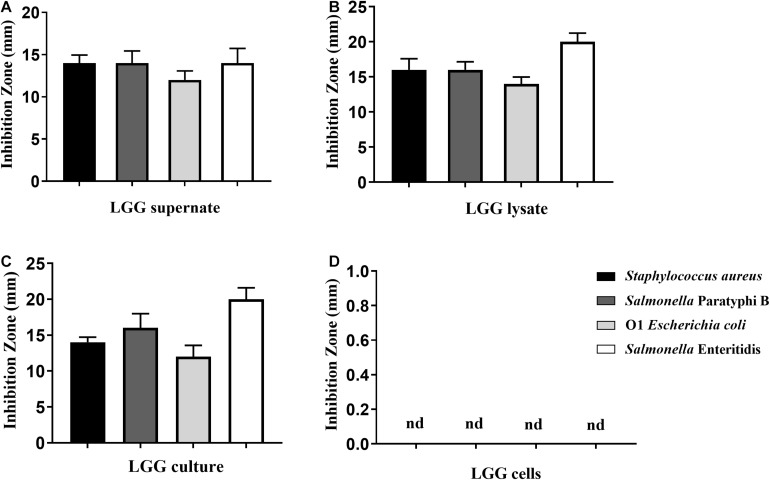
Antibacterial activity of LGG against *Staphylococcus aureus*, *Salmonella* Paratyphi B, O1 *E. coli*, and *Salmonella* Enteritidis. Four pathogenic bacteria were spread onto the LB agar plates. Then **(A)** LGG culture supernatant, **(B)** lysate, **(C)** LGG culture, and **(D)** LGG cells were transferred to holes (5-mm diameter) punched into the agar palates. The antimicrobial activity was determined by the size of the inhibition zone. Bars represent the means ± standard deviations of three independent repetitions. Nd, not detected.

To investigate the antimicrobial activities of LGG, the LGG culture, culture supernatant, lysate, and cells were prepared. The LGG culture, culture supernatant, and lysate showed good inhibitory ability against the growth of *E. coli*, *S. aureus*, *S.* Paratyphi B, and *S.* Enteritidis, and the inhibitory zone was higher than 12 mm (Figures 1A–C). Of these pathogens, the inhibitory effect on *Salmonella* Enteritidis was the best. However, LGG cells could not inhibit the pathogenic bacteria (Figure 1D).

### Effects of LGG on *E. coli* Adhesion to Primary Chicken Intestinal Epithelial Cells

**FIGURE 2:**
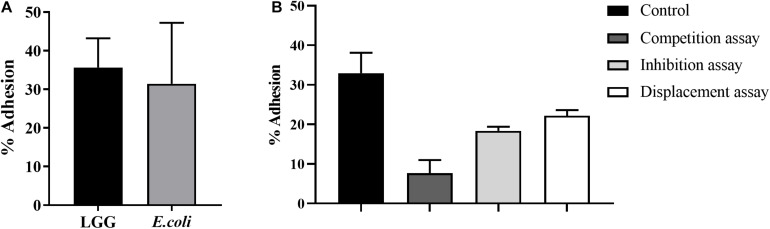
Effects of LGG on *E. coli* adhesion to primary chicken intestinal epithelial cells. **(A)** The LGG and *E. coli* cultures were added to primary chicken intestinal epithelial cells (MOI = 100:1), followed by 1-h incubation at 37°C and 5% CO_2_. Adhesion percentage of LGG and *E. coli* to primary chicken intestinal epithelial cells was expressed as the number of adherent bacteria relative to the total number of bacteria. **(B)** Competition, inhibition, and displacement of *E. coli* adhesion in the presence of LGG. LGG and *E. coli* (1:1) were simultaneously added to primary chicken intestinal epithelial cells for competition assay. In the inhibition assay, LGG was first added to primary chicken intestinal epithelial cells and incubated for 1 h, after which unbound LGG was removed and *E. coli* was added to the wells. On the contrary, in the displacement assay, *E. coli* was added first. Bars represent the means ± standard deviations of three independent repetitions.

One of the important criteria for probiotics is the ability to adhere to the intestinal mucosa. The adhesion levels of LGG and *E. coli* to chicken intestinal epithelial cells were 35.3 and 31.5%, respectively (Figure 2A). In the competition assay, LGG showed the best performance in preventing *E. coli* adhesion to intestinal epithelial cells, as it reduced 75.7% of the *E. coli* adhesion. In the inhibition assay, the *E. coli* adhesion was reduced by 41.7%. In the displacement assay, the adhesion levels of *E. coli* were 22.2% (Figure 2B).

### LGG Promotes Growth Performance and Immunoglobulin

As shown in Table 3, compared to the control group, chickens fed with LGG had significantly increased body weight (*p* < 0.05). On day 21, the average weight of chickens in the experimental group was 423.7 ± 56.6 g, while the average weight of chickens in the control group was 397 ± 54.4 g.

**TABLE 3:** Effects of dietary LGG on body weight and immunoglobulins in chickens.

	Control	LGG
**Average weight gain (g/day)**		
14 days	16.4 ± 2.9^a^	19.0 ± 2.6^b^
21 days	31.2 ± 3.6^a^	33.9 ± 3.1^b^
**Concentration of Immunoglobulins, μg/mL**		
sIgA (14 days)	78.8 ± 7.4^a^	97.0 ± 6.9^b^
IgG (14 days)	429.9 ± 74.7	534.5 ± 47.7
IgM (14 days)	56.9 ± 6.1	54.1 ± 4.3
sIgA (21 days)	68.5 ± 11.0^a^	84.4 ± 12.4^b^
IgG (21 days)	503.0 ± 57.0^a^	726.7 ± 80.1^b^
IgM (21 days)	38.4 ± 9.1^a^	58.1 ± 5.9^b^

*Data are expressed as means ± standard deviations (*n* = 5). ^*a,b*^Values in the same row with different superscripts differ significantly (*p* < 0.05).*

Chickens fed with a diet of 10^6^ CFU g^–1^ LGG had higher IgM, IgG, and sIgA than chickens in the control group. On day 21, the serum concentrations of sIgA, IgG, and IgM in the experimental group were 84.4 ± 12.4, 726.7 ± 80.1, and 58.1 ± 5.9 g/mL, respectively, which were significantly higher than the controls (*p* < 0.05).

### Taxonomic Composition of Intestine Microbiota

**FIGURE 3:**
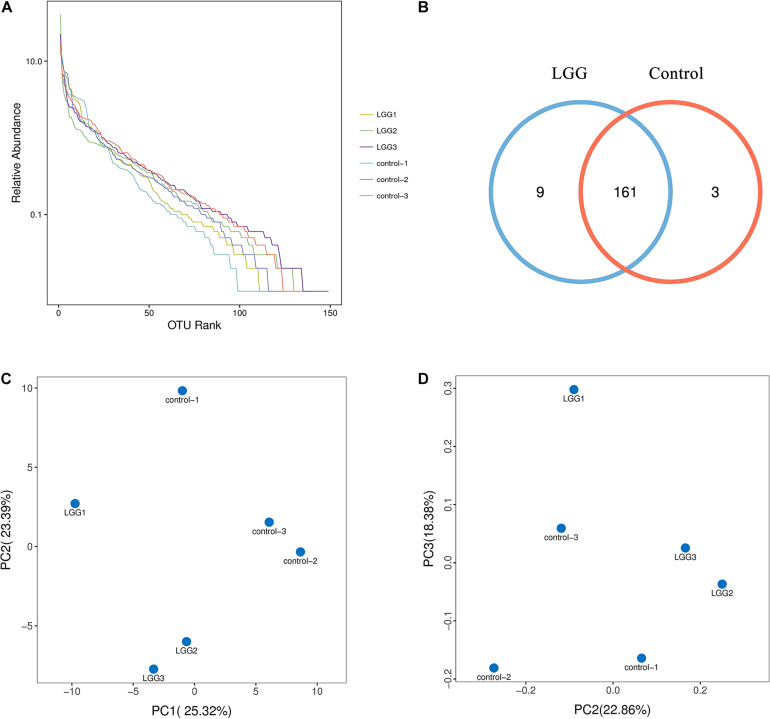
Effects of dietary LGG on cecal microbial diversity in chickens. The cecal contents of chickens fed with LGG and the controls were collected for high-throughput sequence analysis after 21 days of feeding. **(A)** α-diversity of OTU rank abundance. **(B)** OTU Venn diagram between the LGG group and the control group. **(C)** Principal component analysis (PCA) plot based on the distribution of bacterial community. **(D)** Principal coordinates analysis (PCoA) plot using Brary–Curtis distances. The percentage represents the contribution of the principal component to the sample difference.

The 16S rRNA sequence analysis was conducted after 21 days of feeding LGG. The 16S rRNA sequences have been deposited in the Sequence Read Archive (PRJNA699761). As shown in Figure 3A, the OTUs rank abundance indicated that adequate sequence coverage was obtained to reflect the diversity of samples. The species richness of the LGG group was higher than that of the control group. The number of OTU species in the LGG group was 170—more than that in the control group (Figure 3B). The principal component analysis (PCA) and principal coordinates analysis (PCoA) plots showed a good separation of intestine microbiota between the LGG group and the control group (Figures 3C,D). These results indicated that being fed with LGG could increase the diversity of cecum microbiota.

**FIGURE 4:**
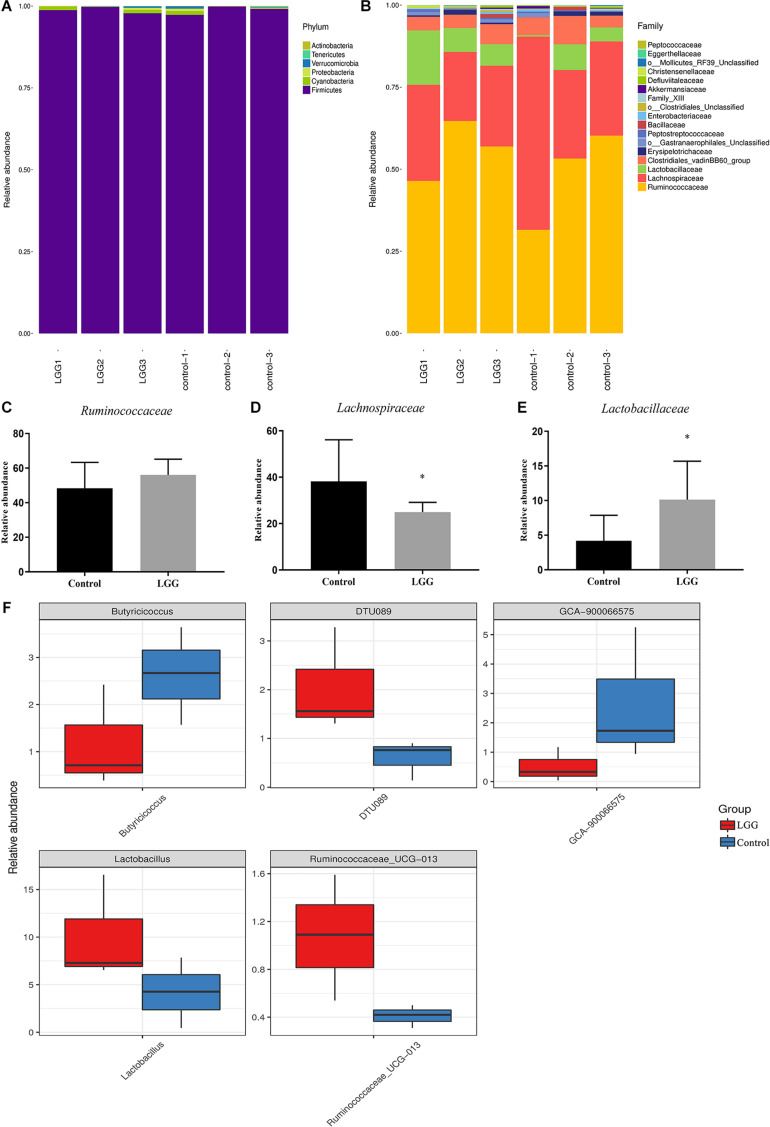
Comparison of identified relative abundance in cecal microbes. Relative abundance of bacterial **(A)** phylum level and **(B)** family level. Relative abundance of **(C)** *Ruminococcaceae*, **(D)** *Lachnospiraceae*, and **(E)** *Lactobacillaceae*. Bars represent the means ± standard deviations of three independent repetitions. *p < 0.05. **(F)** The differential abundance between LGG and the control group based on metastats difference analysis. The abundance distribution of the five species with the greatest difference between the two groups was presented.

As shown in Figure 4A, the main phyla were Firmicutes, Cyanobacteria, and Proteobacteria. After 21 days of LGG feed, the higher percentages at the family level were Ruminococcaceae (56.03%), Lachnospiraceae (24.98%), Lactobacillaceae (10.12%), and the Clostridiales vadinBB60 group (4.8%) (Figure 4B). The relative abundance of Ruminococcaceae and Lactobacillus in chickens fed with a diet of LGG was higher than that in the control group (Figures 4C,E). In contrast, the relative abundance of Lachnospiraceae in chickens fed with a diet of LGG was significantly lower than that in the control group (*p* < 0.05, Figure 4D). The metastats difference analysis showed that the largest differences between the LGG group and the control group were *Butyricicoccus*, *DTU089*, *GCA-900066575*, *Lactobacillus*, and *Ruminococcaceae*_UCG-013. The levels of DTU089, *Lactobacillus*, and *Ruminococcaceae*_UCG-013 in chickens fed with LGG were 0.20, 0.12, and 1.07%, which were all higher than those of the control group. The relative abundance of *Butyricicoccus* and *GCA-900066575* in chickens fed with a diet of LGG was significantly lower than that of the control group (Figure 4F).

### The Expression of Innate Immune-Related Genes Induced by LGG

**FIGURE 5:**
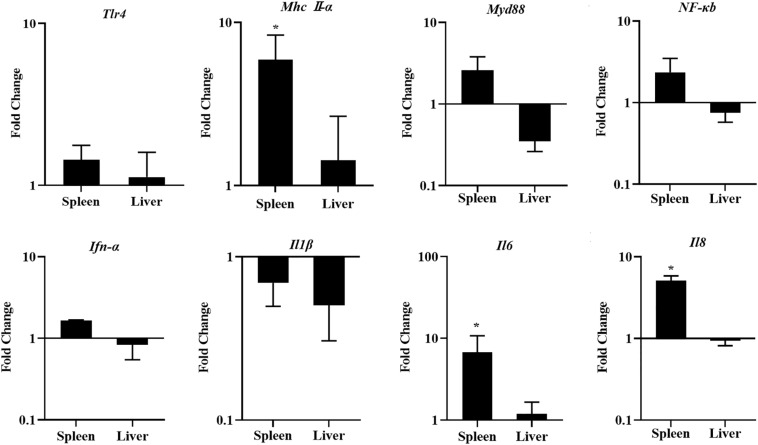
The expression of *Tlr4*, *Mhc II-*α, *Myd88*, *NF-*κ*B*, *Ifn-*α, *Il1*β, *Il6*, and *Il8* in the spleens and livers of chickens after 21 days of feeding. The fold change represents the target gene expression in the diet with LGG compared to the diet of the control group. The relative gene expression levels were normalized to β*-actin*. Bars represent the means ± standard deviations (*n* = 5). *p < 0.05.

To investigate the response of innate immunity in chickens fed with a diet of LGG, the innate immune-related genes were detected in the spleens and livers of chickens fed with LGG compared to the controls after 21 days of feeding. As shown in Figure 5, LGG-supplemented diets upregulated the expressions of innate immune-related genes in the spleens, especially *Mhc II-*α, *Il6*, and *Il8*. However, this induction of innate immune-related genes does not show an obvious change in the liver.

### Survival Rate and *E. coli* Content

**FIGURE 6:**
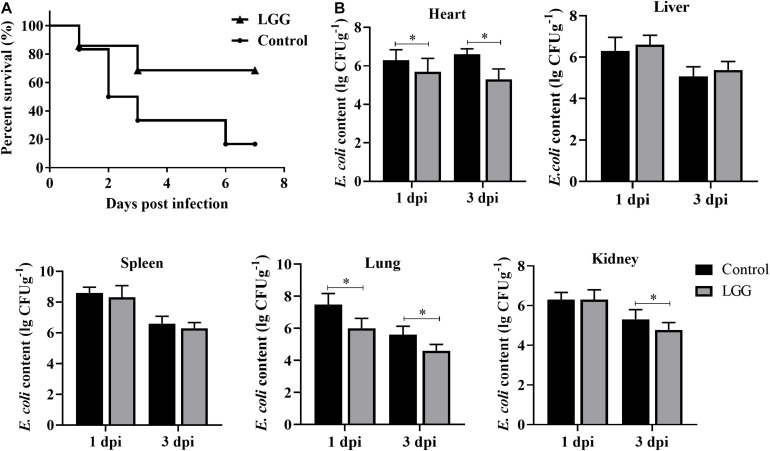
Disease resistance of chickens fed with LGG diet post-challenge with *E. coli*. Chickens were challenged with 10^6^ CFU *E. coli* after 21 days of feeding. **(A)** The survival rate of chickens after infection with *E. coli*. **(B)** *E. coli* content in hearts, livers, spleens, lungs, and kidneys of infected chickens at 1 and 3 dpi (log_10_ CFU g^– 1^). Bars represent the means ± standard deviations (*n* = 5). *p < 0.05.

The survival rate of chickens fed with LGG was higher than that of controls after *E. coli* infection. None of the chickens in the LGG group died by 3 dpi, while the chickens in the control group continued to die until 6 dpi (Figure 6A). The number of *E. coli* was significantly lower in the hearts and lungs of chickens fed with a diet of LGG than in those of the control group at 1 and 3 dpi. However, the *E. coli* content in the livers and spleens showed no difference between the two groups. The *E. coli* content in the kidneys was significantly lower in the experimental group than the control group at 3 dpi (Figure 6B).

### Innate Immune-Related Genes in the Spleen and Liver of the Infected Chickens

**FIGURE 7:**
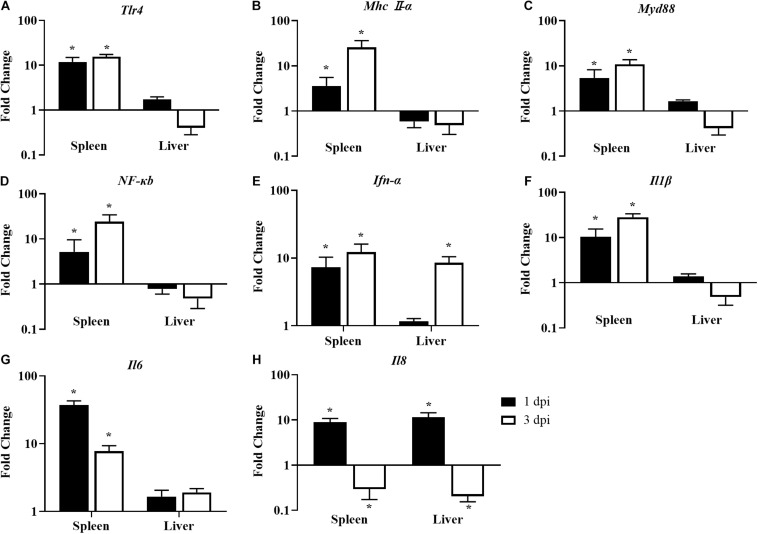
The expression of immune-related genes in the spleens and livers of infected chickens at 1 and 3 dpi. Expression of **(A)** *Tlr4*, **(B)** *Mhc II-*α, **(C)** *Myd88*, **(D)** *NF-*κ*B*, **(E)** *Ifn-*α, **(F)** *Il1*β, **(G)** *Il6*, and **(H)** *Il8*. The fold change represents the target gene expression in the diet with LGG compared to the diet of the control group. The relative gene expression levels were normalized to β*-actin*. Bars represent the means ± standard deviations (*n* = 5). *p < 0.05.

After being challenged with *E. coli*, the innate immune response was significantly activated in the spleens of chickens fed with LGG compared to the controls. The expressions of *Tlr4* and *Mhc II-*α were upregulated by 3.6- to 25.6-fold in the spleen at 1 and 3 dpi (Figures 7A,B). In turn, downstream signal transducing adaptor protein *Myd88* and signaling molecule *NF-*κ*B* were also activated in the spleen at 1 and 3 dpi (Figures 7C,D). Moreover, the expressions of proinflammatory cytokines (*Ifn-*α, *Il1*β, and *Il6*) were significantly upregulated in the spleen in LGG-fed chickens compared to controls at 1 and 3 dpi (Figures 7E–G). The expressions of *Il8* were significantly upregulated in the spleen and the liver at 1 dpi but were significantly downregulated by about 0.2-fold at 3 dpi (Figure 7H).

## Discussion

The important criterion for probiotics is their ability to adhere to intestinal mucosa. Adhesion of *Lactobacillus* to epithelial cells occurs mainly through adhesins, including lipoteichoic acid, S-layer protein, and peptidoglycans (Lönnermark et al., 2012). In previous reports, LGG was demonstrated to be able to adhere to the intestines of infants and adults (Kirjavainen et al., 1998) and to inhibit the adhesion of *E. coli* K88 and *Salmonella typhimurium* to Caco-2 cells (Bogovic Matijasić et al., 2006). Similar to previous research, our results showed that LGG demonstrates good adhesion to primary chicken intestinal epithelial cells. Meanwhile, LGG could reduce the adhesion of *E. coli* to primary chicken intestinal epithelial cells through competition, inhibition, and displacement. Probiotics adhere to and colonize in the intestinal mucous membrane to form a pellicle barrier, which prevents the adhesion of pathogenic bacteria (Fernández et al., 2003; Bogovic Matijasić et al., 2006). *L. rhamnosus* can produce bacteriocin, organic acid, and hydrogen peroxide, which can inhibit the proliferation of pathogens. A previous study showed that *L. rhamnosus* could inhibit the proliferation of *E. coli*, *Salmonella*, and *Clostridium perfringens* (Abhisingha et al., 2018). In this study, LGG cultures, culture supernatant, and lysate showed strong inhibitory effects on *S. aureus*, *E. coli*, *S.* Paratyphi B, and *S.* Enteritidis. Moreover, the inhibition zones of cultures and lysate are 1–5 mm higher than that of the culture supernatant. Based on the above findings, LGG can colonize in the intestinal tract and inhibit the proliferation of some pathogenic bacteria.

It has previously been reported that LGG can promote the weight of weaned pigs (Bocourt et al., 2004). In this study, chickens fed with LGG exhibited a greater average daily weight gain. In another study, dairy infused with LGG and *Bacillus licheniformis* could promote laying rates, egg production, and average weights of eggs (Zhang J. L. et al., 2012). Moreover, a high-throughput sequence analysis of bacterial 16S rRNA showed that feeding chickens with LGG increased the diversity of intestinal microbiota. In chickens fed with LGG for 21 days, the relative abundance of *Lactobacillus* was significantly higher than that in the control group. The lactic acid produced by *Lactobacillus* can inhibit the growth of some pathogenic bacteria (Wang et al., 2019). At the same time, the level of *Clostridium* in chickens fed with LGG was lower. *Clostridium difficile* can produce a variety of exotoxins that have a negative effect on animals (Aschfalk and Müller, 2002).

In innate immunity, the intestinal mucosa is considered to be the first line of defense against pathogen infection. A previous study found less mucus on the surfaces of intestinal mucosa in chickens with less intestinal flora, indicating that intestinal microflora may be involved in establishing the mucosal layer (Forder et al., 2007). Our results indicated that a diet with LGG can regulate intestinal microbiota and maintain intestinal health. In return, the increase in intestinal microbiota diversity and intestinal health can improve growth performance and immune ability (Pereira et al., 2019). In a previous report, LGG was found to decrease the rate of diarrhea in weaned pigs infected with *E. coli* and to upregulate the concentrations of sIgA in the jejunum and ileum (Zhang et al., 2010). Similarly, in this study, a diet with LGG significantly upregulated the concentrations of the immunoglobulins sIgA, IgG, and IgM.

Previous research demonstrated that a diet with probiotics could upregulate the expressions of *Tlr2*, *Tlr3*, *Tlr4*, *Tlr7*, *Tlr8*, and *Tlr10* (Grabig et al., 2006). *Tlr4* is one of the most important pattern recognition receptors that can recognize lipopolysaccharides and heat shock proteins. In turn, *Tlr4* engages *Myd88*, which signals through IRAK4 to turn on a variety of pathways (including *NF-*κ*B*) and induces proinflammatory cytokines. Others have reported that probiotics activate *NF-*κ*B* and stimulate the production of *Tnf-*α *in vitro* (Pagnini et al., 2010). *NF-*κ*B* could induce proinflammatory cytokines such as *Il6*, *Il8*, and *Il1*β, which play a role in the inflammatory response that is responsible for eliminating pathogens. As in previous studies, in this study, a diet with LGG activated *Tlr4*-mediated activation of *NF-*κ*B* and promoted proinflammatory cytokines.

The survival rate of chickens fed with LGG was higher than that of the controls after *E. coli* infection. This result was supported by increased growth performance, immunoglobulin, and enhanced expression of major innate immunity genes, which are involved in the initiation and regulation of immune response against *E. coli*. Most importantly, LGG established antimicrobial properties against various pathogenic bacteria by producing bacteriocin, organic acid, and hydrogen peroxide. In the present study, LGG showed strong inhibition against *S. aureus*, *E. coli*, *S.* Paratyphi B, *S.* Enteritidis, and *E. coli in vitro*. Meanwhile, LGG prevented colonization and persistence of *E. coli* in chicken intestinal epithelial cells. It is also important that LGG can inhibit the pathogenic bacteria by regulating the balance of intestinal microbiota. In addition, the *E. coli* content in the hearts, kidneys, and lungs significantly declined with a diet of LGG after *E. coli* infection. As a result, a diet including LGG can inhibit the proliferation of *E. coli*, enhance the resistance to *E. coli* invasion of the intestinal tract, and improve the disease process *in vivo*. Our results indicated that LGG may be a competitive exclusion approach in terms of controlling bacterial infection.

## Conclusion

In conclusion, LGG showed great probiotic potential, both *in vitro* and *in vivo*. LGG can inhibit the pathogens from adhering to primary chicken intestinal epithelial cells. Chickens fed with LGG exhibited greater growth performance, immunoglobulin concentrations, intestinal health, immune responses, and disease resistance.

## Data Availability Statement

The datasets presented in this study can be found in online repositories. The names of the repository/repositories and accession number(s) can be found below: https://www.ncbi.nlm.nih.gov/sra/?term=PRJNA699761.

## Ethics Statement

The animal study was reviewed and approved by the Committee on the Ethics of Animal Experiments of Yangzhou University.

## Author Contributions

MG designed and investigated the study, and wrote the original draft. CoZ carried out the verification and data analysis. ChZ and XZ discussed the results. YW reviewed and edited the manuscript. All authors contributed to the article and approved the submitted version.

## Conflict of Interest

The authors declare that the research was conducted in the absence of any commercial or financial relationships that could be construed as a potential conflict of interest.
